# Subversion of Innate Defenses by the Interplay between DENV and Pre-Existing Enhancing Antibodies: TLRs Signaling Collapse

**DOI:** 10.1371/journal.pntd.0000924

**Published:** 2010-12-21

**Authors:** Naphak Modhiran, Siripen Kalayanarooj, Sukathida Ubol

**Affiliations:** 1 Department of Microbiology, Faculty of Science, Mahidol University, Bangkok, Thailand; 2 WHO Collaborating Centre Case Management of Dengue/DHF/DSS, Queen Sirikit National Institute of Child Health, Bangkok, Thailand; Pediatric Dengue Vaccine Initiative, United States of America

## Abstract

**Background:**

The phenomenon of antibody dependent enhancement as a major determinant that exacerbates disease severity in DENV infections is well accepted. While the detailed mechanism of antibody enhanced disease severity is unclear, evidence suggests that it is associated with both increased DENV infectivity and suppression of the type I IFN and pro-inflammatory cytokine responses. Therefore, it is imperative for us to understand the intracellular mechanisms altered during ADE infection to decipher the mechanism of severe pathogenesis.

**Methodology/Principal Findings:**

In this present work, qRT-PCR, immunoblotting and gene array analysis were conducted to determine whether DENV-antibody complex infection exerts a suppressive effect on the expression and/or function of the pathogen recognition patterns, focusing on the TLR-signaling pathway. We show here that FcγRI and FcγRIIa synergistically facilitated entry of DENV-antibody complexes into monocytic THP-1 cells. Ligation between DENV-antibody complexes and FcR not only down regulated TLRs gene expression but also up regulated *SARM*, *TANK*, and negative regulators of the NF-κB pathway, resulting in suppression of innate responses but increased viral production. These results were confirmed by blocking with anti-FcγRI or anti-FcγRIIa antibodies which reduced viral production, up-regulated IFN-β synthesis, and increased gene expression in the TLR-dependent signaling pathway. The negative impact of DENV-ADE infection on the TLR-dependent pathway was strongly supported by gene array screening which revealed that both MyD88-dependent and –independent signaling molecules were down regulated during DENV-ADE infection. Importantly, the same phenomenon was seen in PBMC of secondary DHF/DSS patients but not in PBMC of DF patients.

**Conclusions/Significance:**

Our present work demonstrates the mechanism by which DENV uses pre-existing immune mediators to defeat the principal activating pathway of innate defense resulting in suppression of an array of innate immune responses. Interestingly, this phenomenon specifically occurred during the severe form of DENV infection but not in the mild form of disease.

## Author Summary

Dengue is the most common vector-borne viral disease in humans, with 50–100 million infections per year. The severity of dengue ranges from an acute febrile illness, DF, to a life-threatening vascular leakage syndrome with or without shock, DHF/DSS. Determinants of these syndromes are mainly host factors including non protective but cross reactive antibodies which are known as preexisting enhancing antibodies. These antibodies enhance disease severity through increasing the virus infected cell mass and facilitating intracellular virus replication. Here we demonstrate that DENV exploits preexisting subneutralizing antibodies to defeat the pathogen recognition system and to down regulate the TLR signaling pathway resulting in suppression of an array of innate immune responses. Furthermore, we also show that this phenomenon specifically occurs in the severe form of dengue but not in the mild form of disease.

## Introduction

Dengue is the most prevalent vector-borne disease occurring in tropical and subtropical regions with an estimated 50 to 100 million people infected each year. This includes 500,000 cases of life-threatening disease which are dengue hemorrhagic fever (DHF) and dengue shock syndrome (DSS) [1], [2]. Dengue viruses, members of family Flaviviridae, are a group of four genetically distinct serotypes known as DEN-1 to -4. The genome of these viruses is a single-stranded positive sense RNA which is approximately 11 kb in length and encodes for three structural (C, prM, E) and seven non-structural (NS1, NS2A, NS2B, NS3, NS4A, NS4B, NS5) proteins [3].

Infection with dengue virus (DENV) causes two clinically distinct syndromes which are dengue fever, a mild form of the disease and DHF/DSS, a life-threatening disease. The pathophysiology of DHF/DSS development is of interest among researchers since the incidence of DHF/DSS is 25–80 times higher in people previously exposed to DENV than in DENV-naïve individuals, indicating the significance of pre-existing immune mediators such as aberrant T cells, cytokine storms and the enhancing activity of the subneutralizing antibodies [4], [5], [6]. Among these mediators, the presence of enhancing antibodies stand out the most because it is the only risk factor that can explain DHF/DSS development in primary infected infants due to the finding that the peak incidence of DHF/DSS development in infected infants correlates with the decline of maternally derived protective antibodies to non-protective or subneutralizing levels. Moreover, these infants do not experience DHF/DSS accompanying a primary dengue virus infection after the maternally derived antibodies have completely disappeared [7], [8], [9], [10]. This epidemiological data is supported by in vitro enhancement infection experiments in which neat plasma from healthy infants born to dengue-immune mothers enhances dengue virus infection in a manner that correlates with the age-related DHF/DSS development in infants [11], [12]. To further support the role of subneutralizing antibodies, investigators have been able to mimic this phenomenon in a mouse model and in rhesus monkeys [13], [14]. The role of enhancing antibodies in exacerbating disease severity has been reported in other types of infection. For example, *Leishmania* is known to exploit host IgGs facilitating the entry of *Leishmania* amastigotes into macrophages. The entry of amastigotes-antibody complexes via Fcγ receptor ligation does not only allow the numerous parasites to penetrate into macrophages but also suppress the development of cell-mediated immunity resulting in progressive non-healing leishmaniasis in mice [15], [16].

The mechanism by which enhancing antibodies exacerbate dengue disease severity has not been fully established. However, severe dengue is associated with high levels of circulating DENV, and enhancing antibodies have been proposed to facilitate DENV production by at least two mechanisms. Firstly, enhancing antibodies function as a bridge between infectious DENV particles and FcR on cell surfaces resulting in an increased number of infected cells [17], [18]. Interestingly, Rodenhuis-Zybert *et al.* recently demonstrated that enhancing antibodies not only promote entry of the mature DENV but also assists entry of non-infectious virions or immature DENV particles into FcγR bearing cells [19]. Once inside the target cells, these immature viruses replicate effectively. This phenomenon, if occurring in natural dengue virus infections, could significantly contribute to disease severity.

The second mechanism proposed is one in which infection via Fc and FcR ligation switches the intracellular response from an antiviral mode into an immune suppressive mode [20]. This suppression mediates the severity of the secondary dengue virus infection. Thus, to gain more information on the intrinsic role of enhancing antibodies, we further investigated the mechanism of immune evasion induced by DENV-ADE infection.

Once attacked by viruses, host cells immediately recognize the invaders using several types of sensing systems [21]. One of these systems is the Toll-like receptors or TLRs pathway, and six TLRs have been reported to recognize viral invaders. For example, the extracellular TLR-2 and TLR-4 detect viral particles/viral proteins on the cell surface, while the endosomal TLRs recognize viral nucleic acid components such as dsRNA, ssRNA and unmethylated DNA with a CpG motif [22]. Upon ligation to the invader, TLRs trigger a signaling cascade through the recruitment of a set of TIR-domain-containing adaptors including MyD88, TIRAP (MAL), TRIF (TICAM) and TRAM (TICAM2). Based on the MyD88 molecule, the TLR signaling cascade can be divided into two principle pathways, the MyD88-dependent and MyD88-independent (or TRIF-dependent pathway) signaling pathways. While most TLRs trigger the MyD88-dependent signaling pathway via the TIR-containing cytosolic adaptor MyD88, TLR-3 and TLR-4 initiate their signals through TRIF activation [23]. Both MyD88-dependent and TRIF-dependent signaling pathways can activate type I IFN and inflammatory cytokines via NF-κB and the IRFs family [24], [25].

Activation of the TLRs signaling pathway in response to viral infection has been intensively studied [26], [27], [28], [29]. For example, the response against hepatitis C virus infection is mediated by the TLR2 and TLR3 signaling [30], [31], while West Nile Virus (WNV) can be recognized by TLR-3, eliciting an antiviral response shaping innate as well as adaptive immunity in *in vivo* experiments [32], [33]. TLR-3 and TLR-7 have been reported to play important roles in inhibiting dengue virus infection in U937 and HEK293 cells, respectively [34], [35].

The present study investigated the effect of DENV-antibody complex infection on TLR-dependent signaling in a monocytic cell line. The experiments were conducted *in vitro* and *ex vivo*, meaning that infected THP-1 cells and PBMCs from infected patients were used, respectively. This is the first study to show the negative effect of enhancing antibodies on the expression and function of the antigen recognition pathway in human monocytic cells. Results showed that preexisting subneutralizing antibodies were able to ligate infectious DENV particles to both FcγRI and FcγRIIa. Upon ligation, activation of TLR-negative regulators and down-regulation of membrane as well as cytoplasmic TLRs was pronounced, resulting in suppression of TLR-dependent immune activation. These results were also found in secondary DHF PMBC but not in secondary infection DF PBMC.

## Methods

### Ethics statement

The protocol for patient enrollment and sample collection is approved by The Committee on Human Rights Related to Human Experimentation, Mahidol University, Bangkok, Thailand. Dengue-infected patients, which hospitalized at Queen Sirikit National Institute of Child Health, Bangkok, Thailand, were enrolled to the study after the parents/guardians have giving written informed consent. All clinical investigation must have been conducted according to the principles expressed in the Declaration of Helsinki.

### Clinical sample

The enrolled patients were 5–10 years of age. Blood samples were obtained twice, once on the day of admission (fever day) and the other on 30 days after admission (convalescence day). Plasma and PBMCs were separated immediately and were kept frozen at −80°C until required. The patient's disease severity was graded as DF or DHF according to WHO criteria. All enrolled cases were classified as secondary infection by haemagglutination inhibition (HI) titre and IgM ELISA assay [36].

### Virus and cell culture

#### Virus

DENV-2 strain 16681 was used in this study. Virus was propagated in LLC-MK2 cells and kept at −80°C. The titer of stock virus was determined by plaque assay on LLC-MK2 cells [37].

#### Cell culture

THP-1 cells were obtained from ATCC and were cultured in Iscove's modified Dulbecco's medium (IMDM) supplemented with 10% fetal bovine serum (Gibco, USA) at 37°C in a 5% CO_2_ atmosphere.

### Enhancing antibodies

Convalescent serum from a patient infected with DENV serotype 3 (DENV-3) at a 1∶ 10,000 dilution was used in all DENV-ADE infection experiments [38].

### ADE infection in THP-1 cells

Antibody-dependent enhanced infection of DENV-2 16681 into THP-1 cells was conducted as described [38]. Briefly, THP-1 cells were infected with a complex between DENV-2 16681 and the enhancing antibodies at the MOI of 0.01 pfu/cell. After an hour of incubation at 37°C, cells were washed and were further cultured in growth medium. The infected cells and supernatants were harvested at 3, 6, 12, 18, 24 hours and every 24 h for 3 consecutive days. In this experiment, sets of control were performed which were THP-1 cultures infected with DENV at the MOI of 5.0 and 10.0 pfu/cell, THP-1 cells infected with UV-treated-DENV-Ab complexes and the mock-infected THP-1 cells.

### Blocking FcγR using FcγRI (CD64) and FcγRIIa (CD32) antibody

THP-1 cells were pre-incubated at 37°C for 90 minutes with either an anti-human FcγRI MAb or an anti-human FcγRIIa MAb (R&D System, Inc., Minneaspolis, MN) or both antibodies, at a concentration of 5 µg/ml of each antibody. After incubation, cells were washed with IMDM before being infected with DENV or DENV-antibody complexes.

### Viral RNA copy-number titration by fluorogenic real-time RT-PCR or qRT-PCR

RNA was extracted from supernatants using TRIZOL (Invitrogen, CA, USA). The purified viral RNA was then monitored by real time RT-PCR using QuantiTect Probe RT-PCR (Qiagen, Germany) as described by Houng *et al* [39]. Real time RT-PCR amplification, data collection and analysis were performed using a Roter-Gene™ 3000 (Corbett Research, Sydney, Australia). The RNA copy number was calculated using dengue serotype-specific copy standards.

### Oligonucleotide microarray analysis

Total RNA was extracted from infected cells using the QIAgen RNAeasy Kit (QIAgen, Germany). Biotin-UTP labeled cRNA probes were synthesized using 3 µg of cDNA amplified from the total RNA template (Superarray Inc., Frederrick, MD, USA). The labeled cRNA (6 µg) from each sample was then incubated with Oligo GEArray Human Toll-Like Receptor Signaling pathway (OHS-018.2) membranes containing 113 TLR-related genes. The hybridized membranes were washed and hybridization signals were detected using a chemiluminescent system according to the manufacturer's instruction. The intensity of hybridization was determined by ImageMaster TotalLab version 2.00 (Amersham Pharmacia, England) and was acquired in TIFF format. The digital TIFF image files were then analyzed by ClonTech AtlasImage software, version 2.7 (CloneTech, CA, USA). The background was automatically subtracted and standardization of all the signals was performed by normalizing the raw data with β2-microglobulin (β2-m) and Glyceraldehyde 3-phosphate dehydrogenase (GADPH). The correlation between two data sets was tested using Pearson's correlation with *P*-value<0.05. Two-fold and 0.5 fold difference in expression between normalized gene intensities compared between control, DENV, and DENV-ADE samples were considered as significant up-regulation and down-regulation, respectively.

### Quantitation of gene expression levels by real time RT-PCR

The qRT-PCR was used to investigate levels of gene expression. In brief, RNA was extracted from harvested cells using the QIAgen RNAeasy Kit (QIAgen, Germany) and then subjected to first-strand cDNA synthesis before amplification by qRT-PCR using specific primers. The primer sets for *TLR-3*, *TLR-4*, *TLR-7*, *TRIF*, *TRAF-6*, *TRAM*, *IRAK-4*, and *ACTIN* are : *TLR-3*: forward,5′-AGG AAC TCC TTT GCC TTG GT-3′ ; reverse, 5′ – TTT CCA GAG CCG TGC TAA GT- 3′; *TLR-4*: forward, 5′ –TGG ATA CGT TTC CTT ATA AG- 3′ ; reverse, 5′ –CAA GTA CAA GCA AAG TCA TTC- 3′ ; *TLR-7*: forward, 5′ –CCT GGA AAC TTT GGA CCT CA- 3′ ; reverse, 5′-CCA CCA GAC AAA CCA CAC AG- 3′ ; *TRIF*: forward, 5′ – CCC TGT GGA CAG TGG AAG AT- 3′ ; reverse, 5′ –CAA GAC CCT TCA CCC AGA AA- 3′; *TRAF-6*: forward, 5′-GTT GCT GAA ATC GAA GCA CA- 3′; reverse, 5′ –CGG GTT TGC CAG TGT AGA AT- 3′ ; *TRAM*: forward, 5′ – GGG TGA TGT TCG TGT CTG TG- 3′ ; reverse, 5′ –ACT GAG GCG CTG AGG TAA AA- 3′ ; *IRAK-4*: forward, 5′ –CCT TTG CCT TCC ATT GTG AT- 3′ ; reverse, 5′ –GTT TTG GCT TAC GGT TCT GC- 3′; *SARM*: forward, 5′ –TTG CCA AGC AGC AAT GTT AG- 3′ ; reverse, 5′ –TCT CCT CCC AAC CAG AAA TG- 3′; *TANK*: forward, 5′ –CAG GCA TGC ATG GAT AGA GA- 3′ ; reverse, 5′ –TTC AAG CAG AGG AAC ACA GC- 3′; *Beta-actin*: forward, 5′- CCT GGC ACC CAG CAC AAT-3′ ; reverse, 5′GGG CCG GAC TCG TCA TAC- 3′ The qRT-PCR was carried out using the SYBR system (Invitrogen, Oregon, USA), using actin as a control.

### Semi-quantitation of protein production by immunoblotting

Levels of SARM, TRAF6, IRAK4, TLR7, IKK-α, and Rel-A protein production were semi-quantitated using immunoblotting. The intensity of each specific protein was detected using monoclonal antibodies as previously described [38].

### Detection of cytokine production by ELISA

Level of IFN-β production was quantitated using a PBL Medical Laboratories Kit (Piscataway, New Jersey, USA) according to the manufacturer's protocol.

### Statistical analysis

Values were expressed as mean ± standard deviation (SD) of at least three independent observations. Statistical significance was tested by Student's t-test, One-way ANOVA, as indicated in the legend of figure. *P*-values<0.05 were considered significant.

## Results

### DENV-ADE infection is mediated through both FcγRI and FcγRIIa

**Figure 1:**
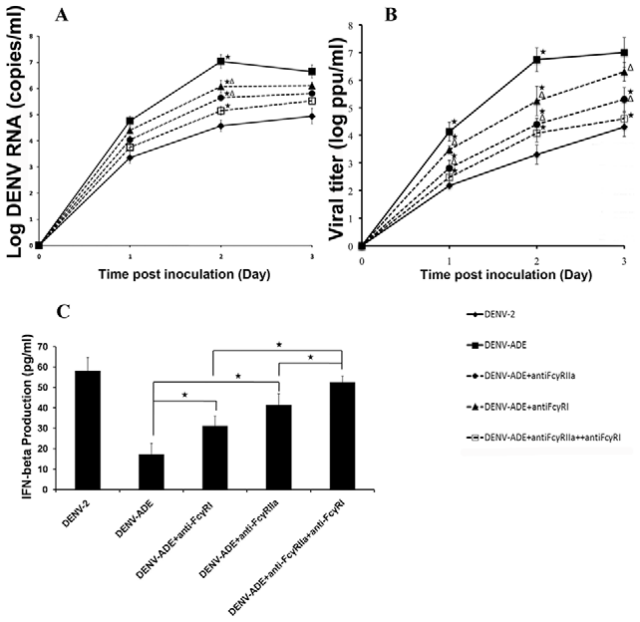
Kinetics of DENV-replication and IFN-β production in THP-1 cells infected with DEN-2 or DENV-Ab complexes. THP-1 cells were pretreated with anti-FcγRI or/and FcγRIIa antibody before being infected with DENV-2 or DENV-enhancing antibody complexes. Supernatants were harvested every 24 h. post inoculation and were subjected to DENV RNA synthesis, infectious virion production and IFN-β production using qRT-PCR, plaque assay and ELISA, respectively. (A and B) Levels of viral RNA synthesis and infectious virus production. Three independent experiments were performed and results are expressed as mean ± SD. Asterisk indicates significant differences between DENV-ADE infected cultures with or without anti-FcR antibody pretreatment at *P*≤0.05. Triangle indicates the significant differences between DENV-ADE infected cultures pretreated with anti-FcγRI or with anti-FcγRIIa at *P*≤0.05. The significant differences were tested using ANOVA analysis, SPSS program. (C) Quantitation of IFN- β production by ELISA. Supernatant fluids harvested at 24 hr. of infection were quantitated for IFN-β production. Asterisk indicates signicficant differences in IFN-β production from DENV-ADE infected cells pretreated with anti-FcR antibodies or mock pretreatment. The P values were obtained from ANOVA analysis.

Two types of FcR, FcγRI (CD64) and FcγRIIa (CD32), have been reported by several investigators to participate in the entry of DENV-antibody complexes in *in vitro* systems [17], [40], [41], [42]. In the present study, the synergistic role of FcγRI and FcγRIIa in DENV-ADE infection in THP-1 cells was investigated. THP-1 cells were pretreated with either anti-FcγRI or anti-FcγRIIa antibodies or both before being infected with either DENV or DENV- enhancing antibody complexes. The level of viral production was monitored by assaying RNA copy number and the number of infectious virions using real-time RT-PCR and plaque assay, respectively. As demonstrated in Fig. 1a–b, blocking of FcγRI or FcγRIIa significantly suppressed viral production in DENV-ADE infection of THP-1 cells. The largest reduction in viral production was found in cells pre-treated with both anti-FcγRI and anti-FcγRIIa antibodies, suggesting that FcγRI and FcγRIIa synergistically mediate the entry of DENV-enhancing antibody complexes into THP-1 cells.

Our previous report demonstrated that DENV-ADE infection significantly suppresses IFN-β production in THP-1 cells (20). Therefore, the level of IFN-β production was used as a marker to test the synergistic role of FcγRI and FcγRIIa on DENV-ADE infection. THP-1 cells were pretreated with anti-FcγRI and anti-FcγRIIa before being infected with DENV-enhancing antibody complexes, and the production of IFN-β was assayed at 24 hr of infection using ELISA. As shown in Fig. 1c, blocking of ADE-infection via FcγRI and FcγRIIa completely restored IFN-β production. Taken together, these results show that DENV-enhancing antibody complexes use both FcγRI and FcγRIIa for entry.

### ADE-infection suppresses TLR-dependent signaling pathways in THP-1 cells

**Figure 2:**
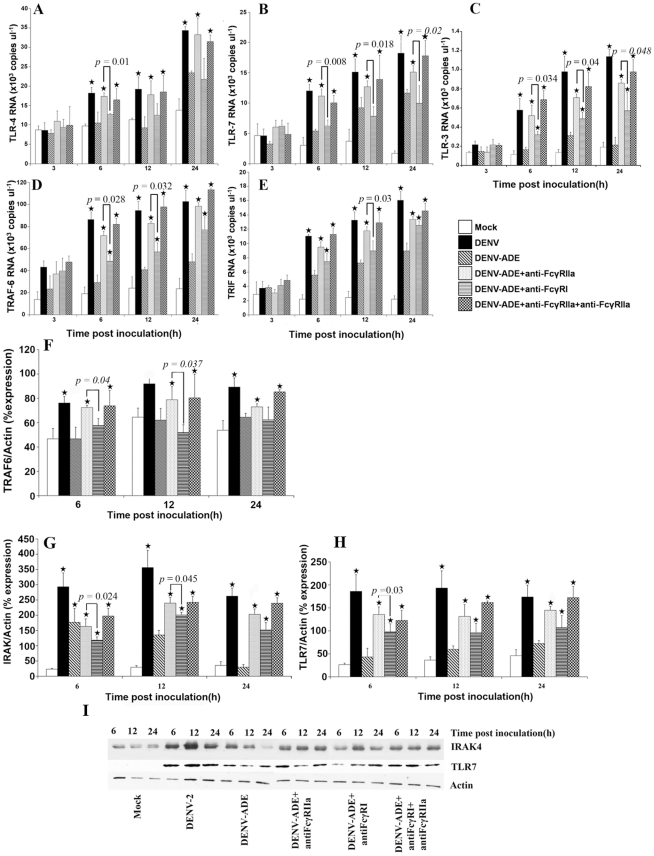
Expression levels of TLRs and TLR-signaling molecules during DENV or DENV-Ab complexes infection. THP-1 cells were pre-treated with either anti-FcγRI antibody or anti-FcγRIIa antibody or both before being infected with DENV or DENV antibody complex. RNA was extracted from harvested cells at the indicated times and RNA copy numbers of (A) *TLR-4*, (B) *TLR-7*, (C) *TLR-3*, (D) *TRAF-6* and (E) *TRIF* were quantified using qRT-PCR. Three independent experiments were performed and results are expressed as mean ± SD. Asterisk indicates significant differences between DENV-ADE infection, DENV infection, DENV-ADE infected cells pretreated with anti-FcγRI antibody or anti-FcγRIIa antibody or pretreated with both antibodies, at *P*≤0.05 using SPSS, ANOVA analysis. Significant differences between DENV-ADE infected cells pretreated with anti- FcγRI and anti-FcγRIIa antibody at the indicated P values were tested using Student's *t*-test. Figure 2 (F–I) are TRAF6, IRAK4 and TLR7 protein production detected by immunblotting using specific monoclonal antibody.

We previously reported that one of the intrinsic roles of ADE-infection is suppression of type I interferon via the RIG-I/MDA-5 signaling pathway [20]. Since type I interferon production is also activated via the TLR pathogen recognition pathway [43], we therefore investigated whether DENV-ADE infection has any effect on TLR expression and/or the TLR-dependent signaling pathway. To answer this question, THP-1 cells were infected with either DENV alone or infected with DENV- enhancing antibody complexes. The expression of a surface membrane TLR (*TLR-4*), endosomal TLRs (*TLR-3*, *TLR-7*) and TLR-signaling molecules (*TRIF*, *TRAF-6* and IRAK4) were monitored using qRT-PCR and immunoblotting. As illustrated in Fig. 2, THP-1 cells infected with DENV-2 significantly stimulated *TLR-3*, *TLR-4*, *TLR-7*, *TRIF* and *TRAF-6* expression. This data correlated with the level of IFN-β production as shown in Fig. 1c. In contrast, DENV- enhancing antibody complex infection significantly suppressed TLR and TLR-signaling molecules in comparison to infection by DENV alone. This data is supported by anti-FcγRI and anti-FcγRIIa treatment in which blocking of DENV-ADE infection via these two receptors restored the expression of TLR(s) and TLR-signaling molecules. This data is also supported by the increased IFN-β production as shown in Fig. 1c. Collectively, DENV-ADE infection could interfere with TLR-dependent signaling via FcγRI and FcγRIIa ligation which corresponded to the reduction of IFN-β production. In addition, pre-treatment with anti-FcγRIIa antibodies restored a higher level of TLRs and TLR-signaling molecules than pretreatment with FcγRI (Fig. 2).

To ensure that phenomenon found in this study is not due to the effect of higher level of viruses produced during ADE-infection mode, control experiments were preformed. THP-1 cultures were infected with DENV at the MOI of 5.0 and 10.0 pfu/cell, or with UV-treated-DENV-enhancing Ab complexes, or were mock infected. THP-1 cultures infected with the MOI of 10.0 replicated DENV to the same level as ADE-infected mode. In contrast, infection by higher MOI of DENV activates stronger TLR-3 and -4 expressions (Figure S1). THP-1 cells infected with UV-DENV-Ab complexes revealed no suppressive effect on IFN-β production (data not shown).

Taken together, this information indicated that suppression of TLRs and TLR signaling pathway demonstrated in our study is due to the infectious immune complexes infection.

### DENV-ADE infection but not DENV-infection activates negative regulators of TLR-signaling pathway in THP-1 cells

**Figure 3:**
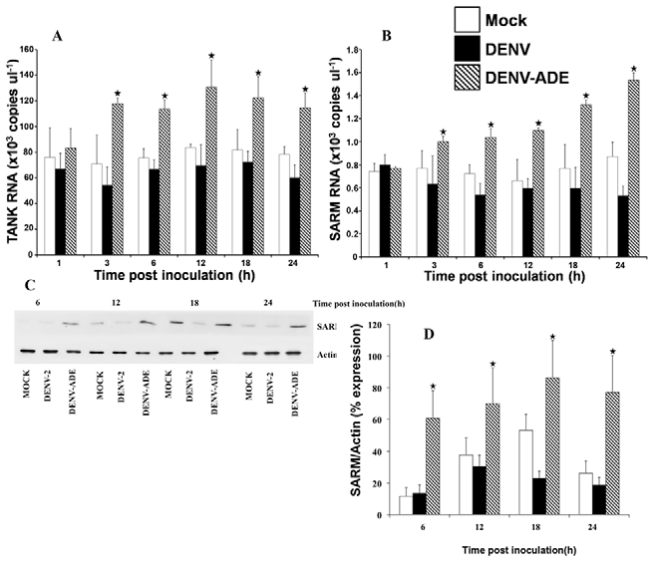
Activation of *TANK* and *SARM* during DENV and DENV-Ab infection. THP-1 cells were infected with DENV alone or with DENV enhancing antibody complexes. The expression levels of (A) *TANK* and (B) *SARM* were quantified by qRT-PCR at various time points. Three independent experiments were performed and results are expressed as mean ± SD. Asterisk indicates the significant difference between DENV-ADE infection and DENV infection at *P*≤0.05. (C–D) detection of SARM at the protein level using immunoblotting.

Suppression of the TLR-dependent signaling pathway may due to down-regulation of TLR synthesis and/or blocking of TLR-signals. Unfortunately, negative regulators of TLR synthesis have not yet been identified. Thus we investigated whether or not down-regulation of the TLR-dependent signaling pathway is due to DENV-ADE infection activating negative regulators of TLRs signaling such as *SARM* and *TANK* and so the levels of *SARM* and *TANK* gene expression were investigated. As shown in Fig. 3, expression levels of *SARM* and *TANK* were significantly increased at 3 hr post DENV-ADE infection, but not in DENV infection.

### Suppression of TLR-dependent signaling pathway in secondary DHF patients

**Figure 4:**
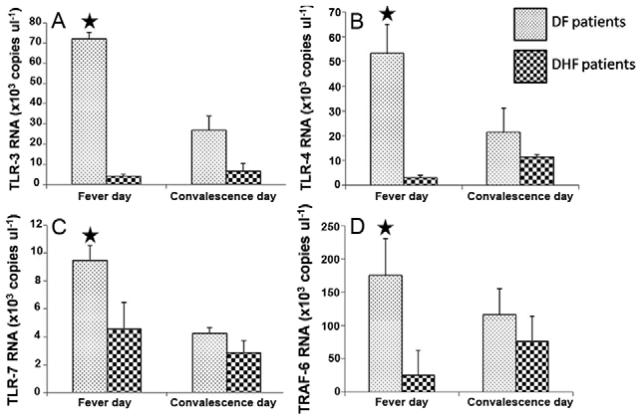
Suppression of the TLRs-dependent signaling pathway during natural DENV infection. PBMCs obtained from DF and secondary DHF patients (n = 15 for each group) on fever and convalescence days were subjected to monitoring the mRNA copy numbers of (A) *TLR-3*, (B) *TLR-4*, (C) *TLR-7* and (D) *TRAF-6* by qRT-PCR. The expression levels of these genes were expressed as mean ± SD of three independent experiments. Asterisk indicates significant differences at *P*≤0.05 using ANOVA analysis.

Since THP-1 is a monocytic cell line it may not be an ideal physiological model of the natural response during DENV infection. Therefore, to confirm the phenomenon found in THP-1 cells, expression levels of *TLR-3*, *TLR-4*, *TLR-7* and *TRAF-6* in PBMCs obtained from secondary DF and secondary DHF patients, on fever and convalescent days were determined by qRT-PCR. Interestingly, expression of these genes was significantly down-regulated in secondary DHF patients but not in DF patients (Fig. 4), suggesting that the TLR-dependent signaling pathway is activated during the mild form of DENV-infection but not in the severe form of the infection.

### TLR and TLR-dependent gene expression profiles in THP-1 cells infected with either DENV or DENV-enhancing antibody complexes

To further elucidate the impact of ADE-infection on TLR-dependent signaling, a TLR-specific oligonucleotide array analysis was conducted to differentiate responses between DENV infection and infection by DENV-enhancing antibody complexes. As shown in Table 1, the expression of 27 out of 113 TLR-related genes was significantly altered during DENV-ADE infection. These genes were categorized on the basis of their functions as TLR, TIR containing adaptor molecules, effector molecules, NF-κB associated molecules, JNK/p38 pathway, IRF pathway, IFN-inducible genes and others. Among these 27 genes, 21 and 6 genes were down- and up-regulated during DENV-ADE infection, respectively. Most of the down-regulated genes are associated with both the MyD88-dependent and -independent signaling pathways such as *TLR-4*, *TIRAP*, *IRAK-2*, *IRAK-4*, *TRIF* (*TICAM1*) and *TRAM* (*TICAM2*). This data indicates that both MyD88-dependent and -independent pathways were suppressed. The expression of TLR-3 and -7 were not included in this table since their expression were suppression less than two folds, 1.7 and 1.6 folds, respectively. Expression of the other types of TLR was undetectable in our array analysis.

**Table 1:** List of genes that expression levels were altered during DENV- or DENV-Ab complex infection.

Genes	Fold changes (mean±SD)		
	DENV-ADE/Mock	DENV/Mock	DENV-ADE/DENV
**Down-regulated genes**			
**TLRs**			
Toll-like receptor 4 (***TLR-4***)	3.18±1.12	11.14±3.2	0.29±0.04
**TIR domain containing adaptors**			
Toll-interleukin 1 receptor (TIR) domain containing adaptor protein (***TIRAP***)	1.23±1.17	2.49±1.31	0.49±0.08
Toll-like receptor adaptor molecule 2 (***TICAM-2***)	29.97±5.40	64.35±22.05	0.47±0.09
Toll-like receptor adaptor molecule 1 (***TICAM1***)	1.19±0.30	26.85±1.99	0.44±0.09
**Effector molecules**			
Interleukin-1 receptor-associated kinase 2 (***IRAK-2***)	1.35±0.18	5.55±0.72	0.24±0.04
Interleukin-1 receptor-associated kinase 4 (***IRAK-4***)	35.16±2.56	82.49±2.43	0.43±0.02
Mitogen-activated protein kinase kinase kinase 7 interacting protein 1 (***MAP3K7IP1***)	1.63±1.38	3.68±1.74	0.44±0.09
Mitogen-activated protein kinase kinase kinase 7 interacting protein 2 (***MAP3K7IP2***)	9.25±3.84	42.65±26.95	0.22±0.04
Ubiquitin-conjugating enzyme E2N (UBC13 homolog, yeast) (***UBE2N***)	5.55±0.36	17.10±0.35	0.32±0.03
TNF receptor-associate d factor 6 (***TRAF6***)	1.96±0.45	4.42±2.32	0.09±0.04
Mitogen-activated protein kinase kinase kinase 7 (***MAP3K7***)	0.51±0.34	2.76±0.56	0.18±0.01
**NF-κB signaling molecules**			
Nuclear factor of kappa light polypeptide gene enhancer in B-cells 2 (p49/p100) (***NFκB2***)	6.42±0.91	16.90±0.35	0.38±0.12
Conserved helix-loop-helix ubiquitous kinase (***CHUK***)	98.12±2.00	225.20±48.17	0.43±0.09
V-rel reticuloendotheliosis viral oncogene homolog A (***RELA***)	4.98±0.90	16.20±8.06	0.28±0.12
V-rel reticuloendotheliosis viral oncogene homolog B (***RELB***)	3.32±0.07	7.43±0.30	0.44±0.01
**JNK/p38 pathway**			
Mitogen-activated protein kinase kinase kinase kinase 4 (***MAP2K4***)	0.61±0.03	1.24±0.94	0.49±0.02
**IRF pathway**			
Interferon regulatory factor 1 (***IRF-1***)	0.81±0.57	5.26±0.25	0.15±0.03
**IFN inducible genes**			
Protein kinase, interferon-inducible double stranded RNA dependent activator (***PKRRA***)	26.28±0.23	68.40±1.20	0.38±0.12
Peroxisome proliferator-activated receptor alpha (***PPARA***)	4.43±0.28	14.08±0.47	0.31±0.11
**Up-regulated genes**			
**NF-κB pathway**			
Inhibitor of kappa light polypeptide gene enhancer in B-cells, kinase beta (***IKBKB***)	3.70±2.61	1.60±1.30	2.54±1.34
Inhibitor of kappa light polypeptide gene enhancer in B-cells, kinase gamma (***IKBKG***)	6.24±0.45	2.38±0.21	3.01±1.76
Nuclear factor of kappa light polypeptide gene enhancer in B-cells inhibitor, epsilon (***NFκBIE***)	2.05±1.05	0.97±0.27	2.11±0.82
**Cytokines**			
Interleukin 10 (***IL-10***)	4.44±0.05	1.74±0.02	2.55±1.50
Interleukin 6 (***IL-6***)	81.16±0.93	1.49±0.31	54.46±0.29
**Other**			
Glypican 1 (**GPC1**)	8.45±1.14	4.10±1.16	2.05±1.06

The suppressive effect was also strongly seen in the NF-κB signaling pathway, in which genes required for IκB degradation and NF-κB activation such as *MAP3K7IP1* (*TAB1*), *MAP3K7IP2* (*TAB2*), *TRAF-6*, *UBE2N* and *MAP3K7* were down-regulated. This observation was confirmed by the reduction of NF-κB signaling molecules including the REL complex, NF-κB2, *CHUK* (*IKK-α*) and MAP4K4 while *NF-κBIE*, an inhibitor of NF-κB, was up-regulated. In addition, suppression of the IRF pathway and IFN-inducible genes was also pronounced during ADE-infection. In addition, the expression of SARM gene was 1.5 folds increased while the activation of TANK gene was undetectable during ADE-infection.

Taken together, these data imply that DENV-ADE infection may activate host negative regulators which in turn down regulate the MyD88-dependent, MyD88–independent and NF-κB signaling pathway, supporting the *in vitro* and *ex vivo* experiments described above.

### Validation of cDNA array analysis by qRT-PCR

**Figure 5:**
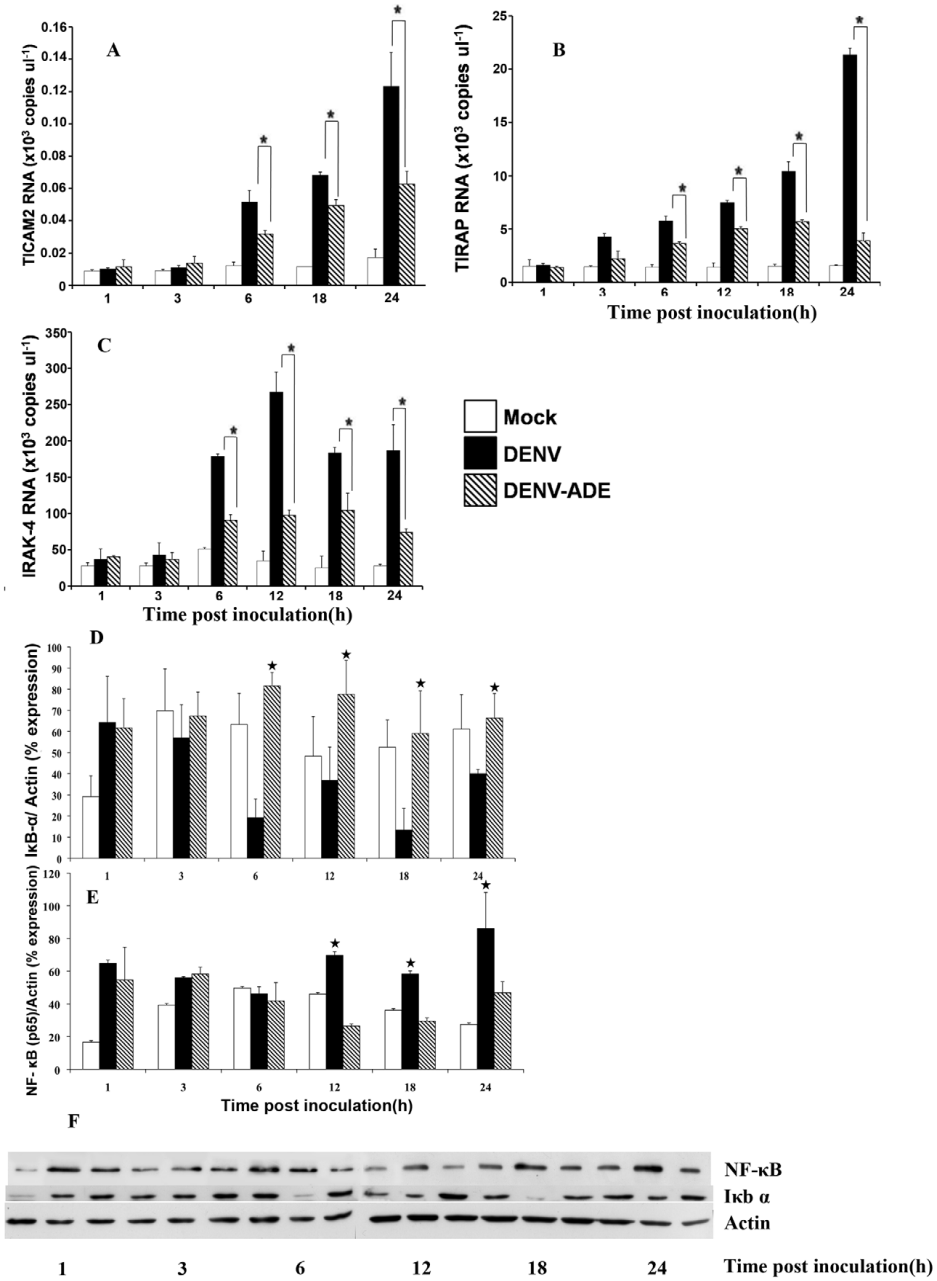
Validation of cDNA array expression data by qRT-PCR and immunoblotting. RT-PCR was carried out using total RNA from THP-1 cells infected with either DENV or DENV-enhancing antibody complexes. The mRNA copy numbers of (A) *TICAM2*, (B) *TIRAP* and (C) *IRAK-4* were monitored by qRT-PCR at indicated time points. The level of (D) IκB-α and (E) NF-κB productions were semi-quantified using immunoblotting. Three independent experiments were performed and results are expressed as mean ± SD. Asterisk indicates significant difference at *P*≤0.05, using ANOVA analysis. (F) is the representative of IκB-α and NF-κB western blotting.

To validate data obtained from the oligonucleotide array analysis, qRT-PCR was used to monitor the expression of 3 genes including of *TICAM2*, *TIRAP* and *IRAK-4* at 3, 6, 12, 18, 24 hours post inoculation. The observed copy numbers of these representative genes are shown in Fig. 5a–c. The levels of expression of these genes were significantly down-regulated in THP-1 cells infected with DENV-ADE infection meaning that data from qRT-PCR confirmed the cDNA analysis. In addition, the protein levels of IKK-α and Rel-A were determined using specific monoclonal antibodies. As shown in fig 5 d–e, degradation of phosphorylated IKK-α and suppression of Rel-A production was significant in DENV-ADE infection mode suggesting that immune complexes infection suppesses NF-κB pathway.

## Discussion

Even though the presence of antibodies that enhance dengue viral infectivity has been known since 1977 [44], the mechanism(s) as to how these antibodies increases viral infectivity and exacerbate disease severity is only just being understood. Our reports and others show that enhancing antibodies is not only facilitating virus entry, but also alter the intracellular responses and that the synergism between the extrinsic and the intrinsic roles of enhancing antibodies significantly increases viral burst size and the total virus yield.

The first mechanism by which antibodies enhance DENV infectivity occurs at the plasma membrane. In this process, enhancing antibodies facilitate the interaction between virus particles and the FcR on target cells. This event gives rise to a higher chance of virus penetration resulting in a greater number of infected cells. The significance of Fc and FcR ligation on ADE-infection has been confirmed using genetically engineered antibody variants which can not bind to FcR [45]. These engineered antibodies abrogate ADE-infection and protect mice from ADE-induced lethal challenge. The types of FcR involved in DENV-antibody complex infection have been investigated intensively by several groups of investigators as well as by our group and all agree that both FcγRI and FcγRIIa facilitate ADE-infection in natural DENV target cells and in DENV susceptible cell lines [12], [16], [20], [41]. Moreover, we observed that FcγRIIa enhanced the infectivity in THP-1 cells more efficiently than FcγRI did (Fig 1a and Fig. 2). Our finding is supported by the previous report using FcγR transfected COS-7 cells in which FcγRIIa enhances dengue virus immune complex infectivity more efficient than FcγRI. This difference may due to mode of virus-immune complex internalization mediated by these two types of FcR [42]. In the other experimental system, engatement of immune-complexes to FcγRI signals through γ-chain to initiate proinflammatory cytokines production and to transport the complexes to MHC-II mediated antigen presentation while interaction between immune-complexes and FcγRIIa impaires proinflammatory cytokine production and antigen presentation [46]. Whether this phenomenon can be applied to DENV-immune complex infection remains unclear.

**Figure 6:**
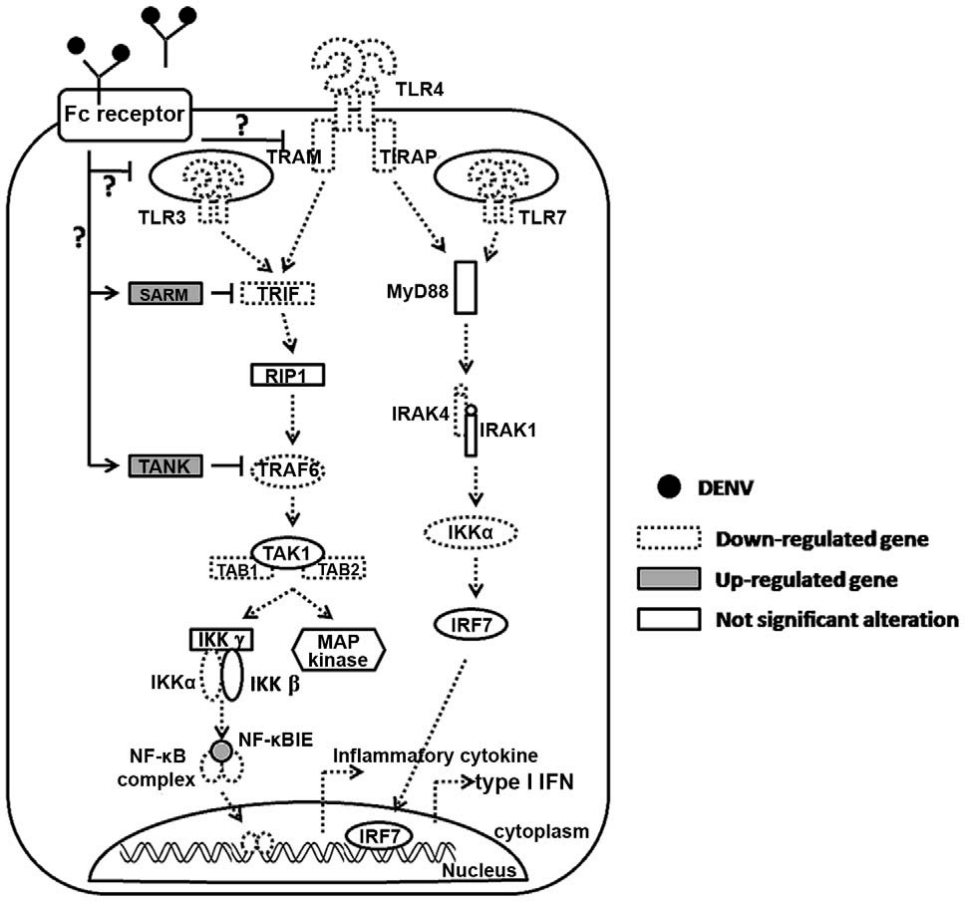
Model illustrating TLR and TLR-signaling pathway collapse during DENV-Ab complex infection. DENV-ADE infection down-regulates TLR-4 and endosomal TLRs synthesis while strongly stimulates *SARM* and *TANK*, negative regulators of *TRIF* and *TRAF-6*, respectively. These events result in suppression of innate responses mediated through the TLR-signaling pathway.

Investigation of the intrinsic role of enhancing antibodies has pointed toward suppression of the innate immune response in which type I interferon and proinflammatory cytokine production are revealed as the main targets [38] and the mechanism of suppression is partly due to ADE infection up regulating negative regulators of the RIG-I/MDA5 signaling pathway [20]. In the present work, we expanded our investigation horizontally to another type I interferon stimulating pathway, the TLR-signaling pathway. Toll-like receptors, some of the most important pattern recognition receptors, are abundant on monocytes/macrophages and dendritic cells, the main *in vivo* target cells for DENV, and TLRs are key players in priming innate responses upon viral infection. They detect invaders and trigger antiviral defenses, interferon and pro-inflammatory cytokines. Interferon then exerts an antiviral activity through activating the JAK/STAT signaling pathway resulting in the activation of interferon stimulated genes which subsequently inhibit viruses by a non-cytolytic mechanism. In turn, invaders can circumvent the interferon response to be able to propagate in the host cell. DNA viruses including hepatitis B virus (HBV) use their envelop and non-envelope proteins to suppress TLRs expression as well as to inhibit responses elicited by TLRs stimulation [47], [48], [49]. The vaccinia virus uses the A46R and A52R proteins to inhibit TLR-signaling molecules such as TRIF, TRAM and IRAK-2 resulting in ablation of type I IFN production [50], [51]. Respiratory syncytial virus (RSV) strain A2 and Measles virus (MeV), a member of the Paramyxoviridae family, can antagonize TLR-7 and TLR-9 induced type I IFN and proinflammatory cytokine production in epithelial cells, hematopoietic cells (T lymphocytes, B lymphocytes, monocytes) and pDC [52]. Similar to A46R and A52R of the vaccinia virus, the NS3-4A heterodimer of Hepatitis C virus inhibits the TLR-3 mediated antiviral response by degrading TRIF while NS5A has been reported to bind directly to MyD88 leading to inhibition of the MyD88-dependent signaling pathway [53], [54]. Moreover, the entire genome of hepatitis C virus has also been found to suppress TLR-3, -4 and -7 in HepG-2 cells [55]. Similar events are also reported during DENV infection. DENV use nonstructural proteins to block phosphorylation and to down-regulate expression of major components of the JAK/STAT pathway causing reduced activation of IFNα/β stimulating genes [56], [57]. All of the antagonists mentioned above are viruses or viral products. However, high-jacking of pre-existing host immune factors by viruses to interfere with the TLR-dependent signaling pathway has not been reported. We are the first group that has been able to show that DENV exploits pre-existing subneutralizing antibodies to defeat the TLRs system. Upon engagement between FcR and DENV-antibody complexes or entry of DENV into monocytic cells via FcR, expression of TLR-3,-4, -7 and TLR signaling molecules were dramatically decreased in parallel to the decreased production of IFN-β. This observation was further confirmed in experiments that showed that production of IFN-β and expression of TLRs were restored when ADE-infected cells were pretreated with anti-FcR antibodies. This data indicates that entry of DENV via FcR preferentially switches off the TLR-dependent IFN stimulating pathway. The switch off mechanism was mediated at the TLRs gene expression level and through activation of the negative signaling regulators, *TANK* and *SARM* (Fig. 6). Unfortunately, the events occurring upstream of TLRs expression and of *SARM* and *TANK* activation are unknown, and therefore require further investigation. However, Kurane and colleagues have demonstrated that functional ITAM is essential for ADE infection [58].

The events shown in Fig. 6 are well supported by the array analysis in which ADE-infection suppressed TLR gene expression and down-regulated the TLR-signaling cascade while several negative regulators of TLR-cascade were up-regulated. Importantly, this phenomenon was also found in natural DENV infection in which TLRs (TLR-3, -4, and -7) and TRAF6 were strongly suppressed in PBMC from secondary DHF patients but not in PBMC of mild disease, secondary DF patients. Taken together, the data obtained from *in vitro* as well as *ex vivo* studies indicate a significant collapse of the TLR-dependent signaling pathway during DENV-enhancing antibody complex infection.

In conclusion, the present study and our previous report on the suppression of TLR-signaling during DENV-ADE infection of THP-1 human monocytic cells clearly show that initiation of infection by DENV-enhancing antibody complexes defeats the major pathogen recognition pattern pathway resulting in suppression of innate antiviral responses. How dengue immune complexes can have such broad effects on cells is not clear. FcRs are well known in their roles in regulating a multitude of innate and adaptive immune responses. After crosslinking by immune complexes, ITAM initiates either negative or positive signals through several types of adaptor molecules such as Syk/ZAP family PTKs, Src family kinase and SHIP-1, SHP-1 etc. [59], [60].The inhibitory activities of SHIP-1, SHP-1 and Lyn/P13k can be can be seen on multiple signaling pathways including TLRs [61], [62]. Even though direct role of these adaptors on RIGI/MDA5 remain unclear but TLRa and RIGI/MDA5 pathways crosstalk at several steps, thus, the negative effect against TLRs possibly block RIGI/MDA5 pathway. Finally, DENV immune complexes formed with neutralizing or partially neutralizing antibodies fail to suppress innate immunity but permit limited infection of monocyte/macrophage resulting in mild disease is crucial problem requiring further study.

## Supporting Information

Figure S1 (A) THP-1 cells infected with DENV alone activates TLRs. THP-1 cells were infected with DENV-enhancing antibody at the MOI of 0.01 pfu/cell or infected with DENV alone at the MOI of 0.01, 5.0 and 10.0 pfu/cell. (B) and (C) Kinetic of replication and the level of TLR-3 and -4 gene expressions were monitored.
(0.15 MB DOC)
