# Supplementary material for: Subversion of Innate Defenses by the Interplay between DENV and Pre-Existing Enhancing Antibodies: TLRs Signaling Collapse
Source: PLoS Negl Trop Dis. 2010 Dec 21;4(12):e924. doi: 10.1371/journal.pntd.0000924 (PMC3006139; doi:10.1371/journal.pntd.0000924)

Supplementary Figure 1. (A) THP-1 cells infected with DENV alone activates TLRs. THP-1 cell were infected with DENV-enhancing antibody at the MOI of 0.01 pfu/cell or infected with DENV alone at the MOI of 0.01, 5.0 and 10.0 pfu/cell. (B) and (C)Kinetic of replication and the level of TLR-3 and -4 gene expressions were monitored.


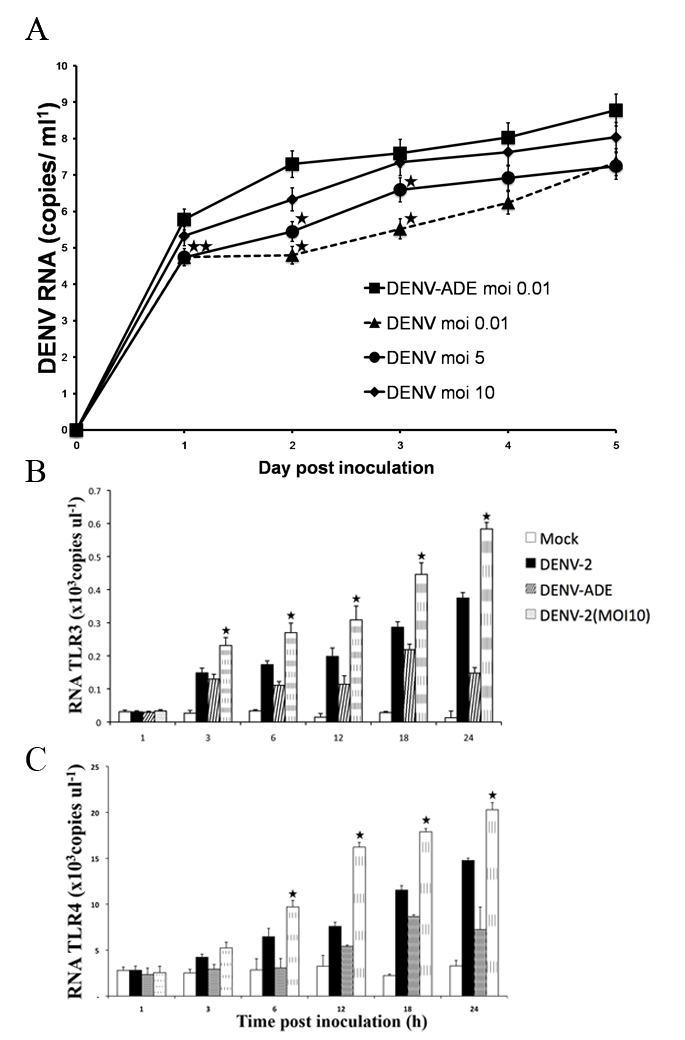

Supplement: Figure S1 — (A) THP-1 cells infected with DENV alone activates TLRs. THP-1 cells were infected with DENV-enhancing antibody at the MOI of 0.01 pfu/cell or infected with DENV alone at the MOI of 0.01, 5.0 and 10.0 pfu/cell. (B) and (C) Kinetic of replication and the level of TLR-3 and -4 gene expressions were monitored. (0.15 MB DOC) [file pntd.0000924.s001.doc]
